# A novel simulation paradigm utilising MRI-derived phosphene maps for cortical prosthetic vision

**DOI:** 10.1088/1741-2552/aceca2

**Published:** 2023-08-10

**Authors:** Haozhe Zac Wang, Yan Tat Wong

**Affiliations:** 1 Department of Electrical and Computer Systems Engineering, Monash University, Melbourne, Australia; 2 Department of Physiology, Monash University, Melbourne, Australia

**Keywords:** cortical visual prosthesis, simulated prosthetic vision, retinotopy, MRI scan, psychophysics

## Abstract

*Objective.* We developed a realistic simulation paradigm for cortical prosthetic vision and investigated whether we can improve visual performance using a novel clustering algorithm. *Approach.* Cortical visual prostheses have been developed to restore sight by stimulating the visual cortex. To investigate the visual experience, previous studies have used uniform phosphene maps, which may not accurately capture generated phosphene map distributions of implant recipients. The current simulation paradigm was based on the Human Connectome Project retinotopy dataset and the placement of implants on the cortices from magnetic resonance imaging scans. Five unique retinotopic maps were derived using this method. To improve performance on these retinotopic maps, we enabled head scanning and a density-based clustering algorithm was then used to relocate centroids of visual stimuli. The impact of these improvements on visual detection performance was tested. Using spatially evenly distributed maps as a control, we recruited ten subjects and evaluated their performance across five sessions on the Berkeley Rudimentary Visual Acuity test and the object recognition task. *Main results.* Performance on control maps is significantly better than on retinotopic maps in both tasks. Both head scanning and the clustering algorithm showed the potential of improving visual ability across multiple sessions in the object recognition task. *Significance.* The current paradigm is the first that simulates the experience of cortical prosthetic vision based on brain scans and implant placement, which captures the spatial distribution of phosphenes more realistically. Utilisation of evenly distributed maps may overestimate the performance that visual prosthetics can restore. This simulation paradigm could be used in clinical practice when making plans for where best to implant cortical visual prostheses.

## 1. Introduction

Promising progress has been made towards restoring the vision of blind people by artificially activating surviving cells in the visual pathway [1–7]. In this study, we focus on cortical visual prostheses which deliver electrical stimulation directly to the visual cortex via implanted electrodes bypassing any damage that might occur at the retina or optic nerve [3, 5, 8–10]. Typically, each electrode elicits a small spot of light in the visual field, called a phosphene [3, 5, 8]. One of the major challenges is to employ available phosphenes to create meaningful patterns that implant recipients can utilise in their day-to-day lives. The perception that current cortical visual prostheses can provide will be rudimentary, with less than 100 phosphenes [3, 5, 8, 11, 12].

Due to the invasive nature of the surgery and implantation of devices, many groups have used computer simulations to investigate the possible outcomes of prosthetic vision primarily for retinal-based prostheses [13–18]. A typical simulation is displayed on a desktop monitor or head-mounted display. Visual stimuli consist of phosphenes which are usually simulated as Gaussian blurred dots. These studies focused on how to provide a functional visual experience by testing different designs, such as the number of electrodes [13, 14, 18], the field of view [16, 19–21] or the image processing pipeline [22–28]. The quality of prosthetic vision has been evaluated using different tasks that mimic daily activities, such as reading [17, 20, 29], object recognition [19, 20, 23, 24, 28], object manipulation [18, 21] or navigation [16, 18, 21, 30].

However, a possible issue in previous simulation studies is that idealised phosphene maps are commonly used. A phosphene map represents the possible locations of phosphenes in the visual field. Many studies assume that implants will generate regular phosphene maps in our visual fields [19, 31, 32]. Phosphenes in these simulations are commonly arranged in a square or hexagonal grid. This issue is compounded for cortical visual prostheses due to the curvature of the visual cortex and irregular retinotopic maps. Some groups have introduced randomisation into phosphene maps [13, 29, 33–36], or presented phosphenes restricted to sections of the visual field [18], however, these simulated phosphene maps may still overestimate the visual ability of implant recipients.

A common cortical visual prosthesis design is to implant electrodes clustered in small arrays [12, 37–39], which results in phosphenes distributed unevenly in the visual field. The differences in cortical surface [40] also affect how surgeons place implants, which will result in a phosphene map that is unique to each recipient. These individual variabilities will mean that the task to present recognisable patterns using phosphenes is more challenging than expected.

This study aims to develop a more realistic simulation paradigm. Several studies confirm that phosphene locations follow the retinotopic maps of the individual [12, 41, 42]. Hence, in our new simulation paradigm, realistic phosphene maps were derived by using retinotopy data and guidance about implant placement from surgeons. This method is generalisable to any implant configuration and surgical decision. An eye tracker was used to lock visual stimuli to participants’ eye positions [43]. Participants’ visual abilities using these maps were evaluated using Berkeley rudimentary vision test (BRVT) and an object recognition task.

We hypothesised that previously used phosphene maps may overestimate the visual ability provided by cortical visual prostheses. This study presents a novel simulation paradigm to improve our understanding of providing functional cortical prosthetic vision. In clinical practice, the simulation paradigm may help neurosurgeons with pre-surgery planning and optimise the visual experience of cortical visual prostheses.

## 2. Methods

### 2.1. Creation of brain-derived retinotopic phosphene maps

**Figure 1.**
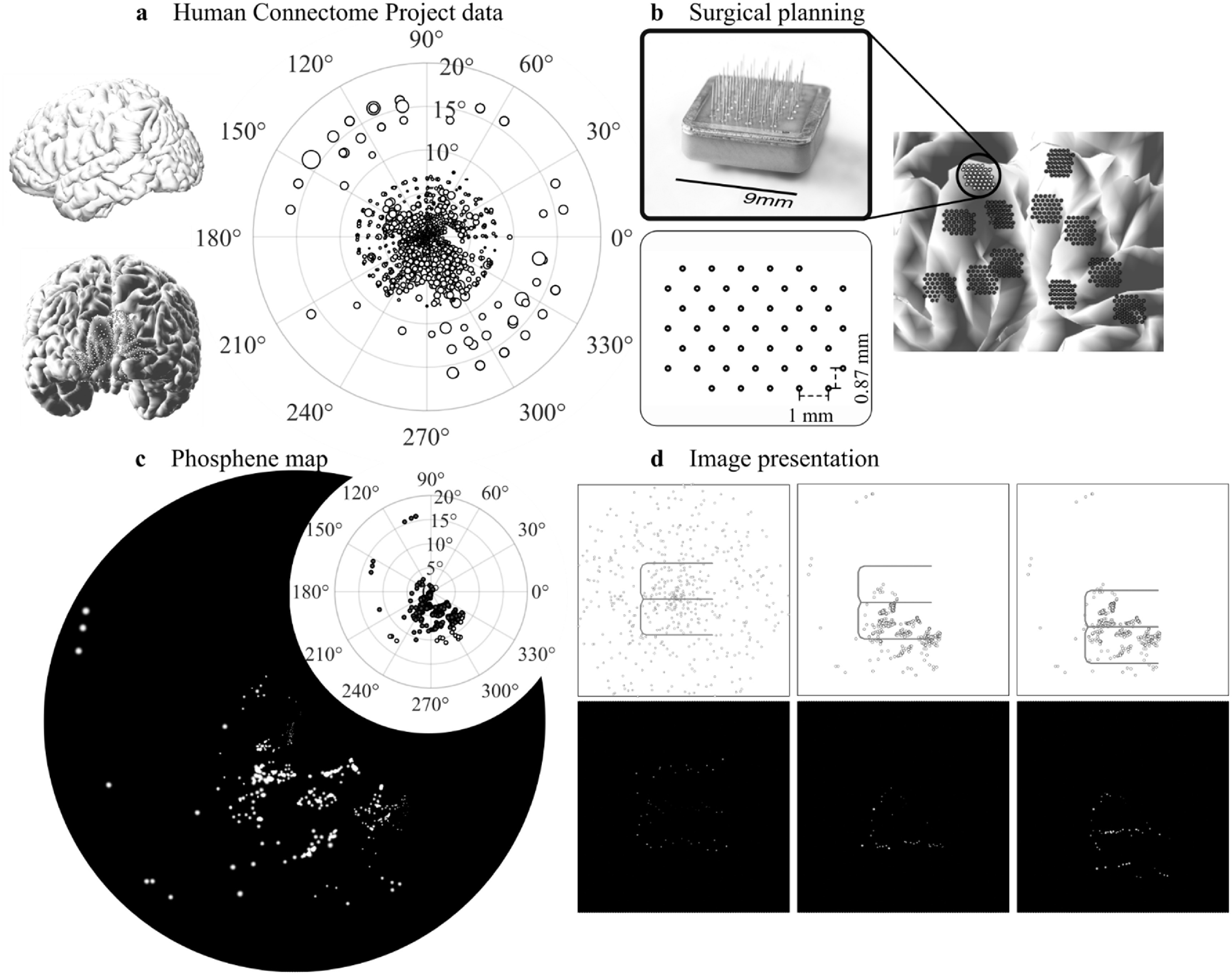
The simulation paradigm. (a) Retinotopic data of one subject from the Human Connectome Project. On the left are the lateral view and posterior view of the brain. On the right are the population receptive fields of V1 and V2 grayordinates. Each white dot on the brain and visual field represents the grayordinate and corresponding population receptive field. (b) Surgical planning by placing implants on the MRI scan of the subject’s visual cortex. On the top left is the Monash Vision Group Gennaris implant, and on the bottom left is the layout of electrodes. On the right is the placement of implants on V1 and V2. (c) Derived phosphene map using the dataset (top right). The image shows the rendered images after adjusting the phosphene size. (d) Image presentation. On the left is the control condition, in the middle is a naïve method (retinotopic condition) where the visual stimulus is placed at the centre of the phosphene map. On the right is a clustering method (retinotopic + clustering condition) where the visual stimulus is placed at a region where the phosphenes are denser.

To create brain-derived retinotopic phosphene maps we used a retinotopy dataset from the Human Connectome Project [44, 45], which contained structural magnetic resonance imaging (MRI) brain scans with associated retinotopic mappings. Implants were placed on the brain scans and the resulting electrode locations were projected onto the visual field (figure 1). Phosphene size was then calculated based on eccentricity of the electrode location on the cortex.

#### 2.1.1. Visuotopic mapping

We used 7 T retinotopy data provided by the Human Connectome Project [44]. The project recruited 181 subjects, where each subject participated in six 5 min population receptive-field mapping runs allowing estimates of population receptive-field position (angle and eccentricity) and population receptive-field size (radius in degrees) for each ‘grayordinate’ (cortical surface vertex or subcortical voxel; figure 1(a)). Each subject’s brain is described using 91 282 cortical vertices and subcortical voxels, with grayordinates spaced on average 2 mm apart. For this study, we randomly chose five subjects’ brains from the dataset. As the phosphene maps depend on the location of the implanted electrodes we chose to utilise the Monash Vision Group Gennaris implant (figure 1(b)) [46], with 43 electrodes spaced 1 mm apart.

#### 2.1.2. Placement of implants

For each brain, we planned the implantation of two, four, and six electrode arrays on each hemisphere (four, eight, and twelve for each brain) with consultation of a neurosurgeon and generated three different phosphene maps. Currently, placing one to two implants in each hemisphere (four implants in total) is achievable for human trials [12] with several cortical prosthetic vision projects proposing to place approximately eight implants on the visual cortex [37, 39]. To achieve a functional visual experience [13, 47], we extended our simulations to 12 implants, which is a surgical challenge but a feasible goal. To place an implant on the cortical surface, we first picked a vertex on the visual cortex and used this vertex as the centre of the electrode array implantation site. We then manually adjusted the electrode array’s angle, so that it was parallel to the cortical surface. Anatomical and hardware constraints were taken into consideration when selecting implantation sites described in the next sections.

##### 2.1.2.1. Anatomical constraints to device implantation.

Implants were placed on gyri whenever possible; sulci were avoided as implanting in these regions can result in incomplete penetration of electrodes and subsequent stimulation failure. We placed most of the electrodes in V1 and V2 as stimulation of the higher-order visual areas would result in a decreased probability of phosphene generation [48]. We used Wang *et al*’s method to define V1 and V2, provided in the Human Connectome Project data set [49]. No implant was placed on the inferior surface or medial surface of the occipital cortex as the surgical process to implant in these regions may concern the sagittal sinus and the potential risk of causing blood vessel damage is high.

##### 2.1.2.2. Hardware and surgical constraints to device implantation

The rotational angles of implants were limited to ±45 ° (relative to the coronal plane) and the distance between the implants and coil centroid needed to be less than 20 mm to ensure the magnetic induction coil to power the implants could operate properly [50]. Hence the medial regions in the longitudinal fissure were excluded, and regions that were too lateral (exceeding V2 boundary) were not ideal. Finally, the electrode array of implants needed to be separated by a minimum distance (3.6 mm) to account for electronics used for stimulation and encapsulation that form the rest of the cortical visual prosthesis. To achieve a functional visual experience [13, 47], we placed six implants on each hemisphere, and due to the limited area of V1 and V2, we allowed electrode arrays to be placed closer together for this number of implants. We quantified overlap by estimating the maximum overlapping distance between the long edges of two neighbouring implants. On average 4.6 implants were affected for each brain, with overlap ranging between 0.64 and 2.1 mm, median = 1.25 mm. Surgical placement of 12 implants is currently surgically not feasible and would require reducing encapsulation size, however, simulation of higher count implant configurations still has value for future planning.

#### 2.1.3. Derivation of phosphene features

After we determined the placement of implants, we utilised the underlying retinotopic maps to generate the phosphene maps. MATLAB was used to visualise and generate the phosphene maps. Since the grayordinates describing the cortical surface were spaced 2 mm apart, but the Monash Vision Group implants have an inter-electrode distance of 1 mm, we needed to estimate the corresponding point on the visual field for each electrode. To do this we used a K-nearest neighbours’ algorithm to find the 50 nearest grayordinates on the cortex closest to the electrode followed by an inverse distance weighting to estimate the coordinates and the population receptive field size based on those 50 grayordinates (figure 1(c)). It was assumed that each electrode would elicit one phosphene [3, 5, 8].

As a control, we utilised phosphene maps that were spatially evenly distributed in the visual field similar to those used by previous studies [13, 18, 29, 34–36]. The number of phosphenes in the control maps was the same as in the brain-derived retinotopic maps. To capture the cortical magnification effect, we generated control maps based on averaged data from Human Connectome Project dataset. We first found the linear relationship between eccentricity and receptive field size for the average data of 181 subjects. We assigned the location for each phosphene randomly and computed the corresponding receptive field size based on the linear fit. The receptive field was used to determine the phosphene size in the next step.

#### 2.1.4. Determining phosphene size

An assumption of this work is that the size of phosphenes corresponds approximately to the size of cortex activated by stimulation and the receptive field sizes of the neurons being activated. Hence, in our simulation we utilised past data that demonstrated how the receptive field size is dependent on eccentricity [51], and phosphene size is related to activated cortical area and eccentricity [52]. By applying a linear model for eccentricity (predictor) and population receptive-field size (response), we found 95% confidence bounds for each response observation and replaced the data points with predicted values if they exceed the boundary. There were two scaling factors needed to estimate phosphene size, to scale the population receptive-field size to phosphene size and then to relate subdural stimulation to intracortical stimulation.

For the first scaling factor, we found a linear fit between eccentricity and population receptive-field size in the data set for each subject and this was then used to relate eccentricity and phosphene size [52]. The activated cortical area was estimated by equation (1), where *AC* is the diameter of the activated cortex, and *I* is the current level. All parameters were taken from the final model in [52], which provides the best prediction result for all cases. *MD* (5.3 mm) is the predicted maximum diameter, *I*_50_ (0.89 mA) is half of the saturation current, and *slope* (5.85 mm mA^−1^) is the maximum slope of diameter increment as the current increases

\begin{equation*}AC = MD/\left( {1 + {{\text{e}}^{\left( { - slope*\left( {I - {I_{50}}{\text{ }}} \right){\text{ }}} \right)}}{\text{ }}} \right).\end{equation*}

The eccentricity was used to determine the cortical magnification factor (*M*) via equation (2), where *e*2 (3.67°) is the eccentricity at which *M* falls to half of the foveal value, and *A* (29.8) is the cortical scaling factor. The values of *I*_50_ and *A* minimise the error between the actual and predicted phosphene sizes, while the value of *MD* maximises the correlation between predicted and actual phosphene size [52]

\begin{equation*}1/M = \left( {Ecc + e2} \right)/A.\end{equation*}

By using *I* = *I*_50_ in equation (1), we could derive the relationship between eccentricity and phosphene size in radius is

\begin{align*}PS&amp; = AC \times 1/M = AC/A \times Ecc + AC \times e2/A\nonumber\\&amp; = 0.044 \times ECC + 0.163.\end{align*}

For example, if we found the relationship between eccentricity and population receptive-field size for one subject is *PS* = *a*× *ECC* + *b*, we would scale this equation by a factor of 0.044/*a* and get

\begin{equation*}PS = 0.044 \times Ecc + b/a \times 0.007.\end{equation*}

However, this has only been tested for subdural electrodes and previous studies have shown that intracortical stimulation would result in smaller phosphenes [5]. This is because the current used for intracortical stimulation is typically lower than subdural electrodes, which will lead to a smaller activated area [12, 52]. Hence, a second scaling factor was utilised.

One way to find this scaling factor is to estimate the activated cortical area for each stimulation method. For subdural electrodes, by using *I* = *I*_50_, the radius was about 1.33 mm. For intracortical electrodes, by assuming *I* = 80 *μ*A [12], the radius was 0.34 mm. Hence the scaling factor would be around 0.34/1.33 = 0.256. By multiplying the two parameters, we can get a scaling factor from population receptive-field size to phosphene size of 0.011/*a*. Each phosphene was simulated as a Gaussian blurred dot with the sigma of the Gaussian filter equal to one-third of phosphenes’ radius (figure 1(c)). The luminance of overlapping phosphenes was summed and capped at the maximum value [53].

### 2.2. Comparing brain-derived retinotopic maps to regular grid maps

#### 2.2.1. Participants

We recruited ten participants to test five different phosphene maps (two participants for each map). All participants had normal or corrected-to-normal vision. The experiments include a visual acuity (VA) test and an object recognition task. For the VA test, we ran five sessions with two-week gaps in between; for the object recognition task, we ran five sessions with a one-week gap in between, four subjects dropped out, and were replaced by newly recruited participants. Six participants completed five sessions and four participants completed three sessions. Each session lasted for approximately one and a half hours. We obtained informed consent from all subjects. The project was approved by Monash University Human Research Ethics Committee (MUHREC Project Number 12525).

#### 2.2.2. VA test

Previous studies have shown that letter acuity metrics are a reliable reference to compare different simulation studies [54]. Letter acuity is also a good predictor of more complex visually guided tasks, such as reading, object recognition and navigation [54]. Since VA tests are usually clinically proven, efficient and provide quantifiable metrics, we decided to use a standardised VA test to evaluate participants’ visual ability based on the BRVT [55, 56]. We utilised the single tumbling E test (0.8–2.6 logarithm of the minimum angle of resolution (LogMAR), step = 0.2 LogMAR) and the grating acuity test (2.3–2.9 LogMAR, step = 0.2 LogMAR). If a participant’s performance was worse than 2.9 LogMAR, we assigned a value of 3 LogMAR. This helped preserve the data without exaggerating the differences between groups. For the single tumbling E test, at each VA level, at least four out of eight trials needed to be correct to indicate that VA is at, or better than the current level. For the grating acuity test, at each VA level, seven out of eight trials needed to be correct to indicate that VA is at, or better than the current level.

For the single tumbling E test, to generate the visual stimulus on a phosphene map, we first resized the E image to corresponding testing VA and skeletonised its stroke to 1 pixel width. For each pixel in the resulting image, we found the nearest phosphene and presented a spot of light at that location. For the grating acuity test, we used direct masking, where we overlapped the phosphene map with the image and presented all the phosphenes under the white regions.

#### 2.2.3. Object recognition task

Object recognition tasks are widely used to evaluate the outcome of prosthetic vision in simulation [19, 20, 23, 24, 28]. These tasks provide a better estimation of the usability of prosthetic vision for daily activities. We used images from the fashion-MNIST dataset that include daily objects. Five categories were used, including tops, trousers, dresses, sneakers and bags. For phosphene rendering, we resized images to 540 × 540 pixels and uses direct masking to present each image on a given phosphene map.

Previous studies have tried to quantify the benefit of head scanning in VA tests [57]. We wanted to further explore this to investigate how head scanning influences more complex tasks, such as object recognition tasks. Using the remote mode of Eyelink 1000, we encouraged participants to perform head scanning on retinotopic maps on one condition. A target sticker is placed on participants’ forehead to capture the head movement. Participants could move their heads to navigate on a 5-by-5 grid where the centre of each cell is 3° apart.

### 2.3. An algorithm to relocate images and the experiment design

Since phosphenes on retinotopic maps tend to cluster into certain regions, a density-based spatial clustering (DBSCAN) algorithm was used to determine the most suitable region to present stimuli. The clustering algorithm can classify each phosphene into a specific group based on the distance between them. Compared to other clustering methods, such as k-mean clustering, spatial clustering is more robust to noise and generates more consistent output [58]. Since we only presented simple visual stimuli, we adjusted parameters to separate the phosphenes into two clusters: the target cluster and outliers. Parameters were kept consistent for all retinotopic maps, where the epsilon was 200 pixels (about 5° in the visual field), and the minimum number of neighbour points was 100. The centres of visual stimuli were shifted to the centroids of the target clusters.

**Figure 2.**
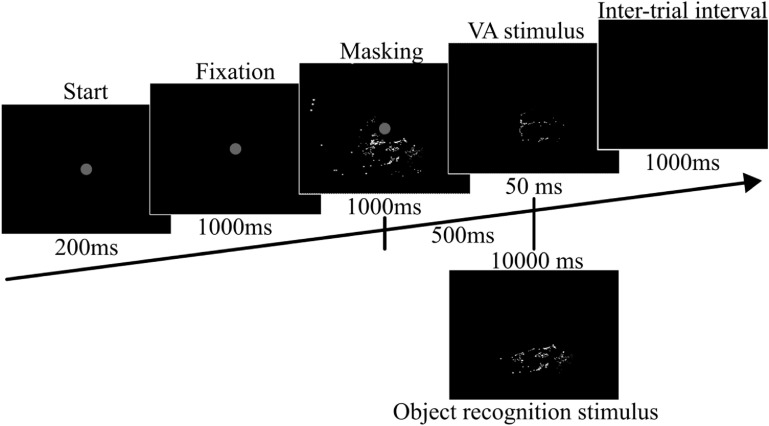
The experimental task. Grey-filled circles indicate fixation points, eye position was captured by Eyelink-1000. Feedback was not provided. All phosphenes of the phosphene map were presented before the stimulus as masking. In addition to limiting the persistence of vision, this helps participants understand the idea that phosphenes of visual stimuli are selected from the phosphene map. The visual stimuli are either from the visual acuity (VA) test or the object recognition task.

Participants were asked to sit in a dark room and respond to visual stimuli with keypresses. All stimuli were displayed on a display++ LCD monitor. Participants were also asked to rest their heads on a chin rest to stabilise their head position, except for the head scanning condition. An eye-tracking device (Eyelink 1000) was used to track eye position, stimuli would only appear when participants fixate on the fixation point in the middle of the screen, as shown in figure 2. For head-stabilised mode, if participants’ eye moves slightly within the tolerant setting, the visual stimuli will move together with eye position. For remote mode, to avoid images vibrating due to changes in head-eye position, we fixed stimuli to the centre of the screen.

For the VA test, each session had two category sets, with each having three conditions (3 × 3 = 9 conditions each session). The first set had different numbers of implants, 4, 8 and 12 corresponding to 172, 344 and 516 phosphenes respectively. The second set had three different ways of presenting visual stimuli. The first method represented visual stimuli in the central visual field using a retinotopic-derived phosphene map. The second method was a control map with a matching number of phosphenes. The third method was similar to the first but used the DBSCAN algorithm to shift the visual stimuli in the visual field and present it in the region where the phosphene density was highest. For each trial, the experimental procedure is shown in figure 2. After each VA test, we ran a series of tests around the measured VA to validate our result. This validation test had three VA levels, VA + 0.2 LogMAR, VA − 0.2 LogMAR and at VA, each level has 12 trials.

For the object recognition task, we used phosphene maps with 12 implants (516 phosphenes). In addition to the three presenting methods, we added a fourth condition where participants can use head scanning on the retinotopic-derived phosphene maps.

### 2.4. Data analysis

For the VA test, participants’ performance was evaluated using two different measurements: the VA and the average response time of the validation test. We did not use response time from the VA test because the test is adaptive to participants’ responses. As the number and types of stimuli were different for each test the response times were averaged within each validation test to match the sample size of VA. For the object recognition task, we used accuracy and response time to evaluate participants’ performance. For VA results, since an unequal variance was observed between conditions, we decided to use a linear mixed model to test whether group differences in means are significant. Bonferroni correction was used for pairwise comparison. For the object recognition task, an ANOVA test followed by Tuckey *post hoc* analysis was used. Data from the first two sessions were not included in the ANOVA test since an obvious learning effect was observed in retinotopy, clustering and head scanning group. The learning effect was measured by averaging VA (or accuracy) and response times for each session and each participant as well as each presentation method. MATLAB 2021a and SPSS version 29.0.0.0 were used for data analysis.

Finally, we aimed to find a metric that could predict how ‘good’ a phosphene map is or alternatively how well can a visual stimulus be presented on a phosphene map. Participants’ performance was evaluated using VA. We used three different metrics: the complexity of phosphene maps [59], the mutual information between phosphene maps and stimuli, and the area under the phosphene map.

## 3. Results

### 3.1. Participants perform worse with MRI-derived retinotopic maps

**Figure 3.**
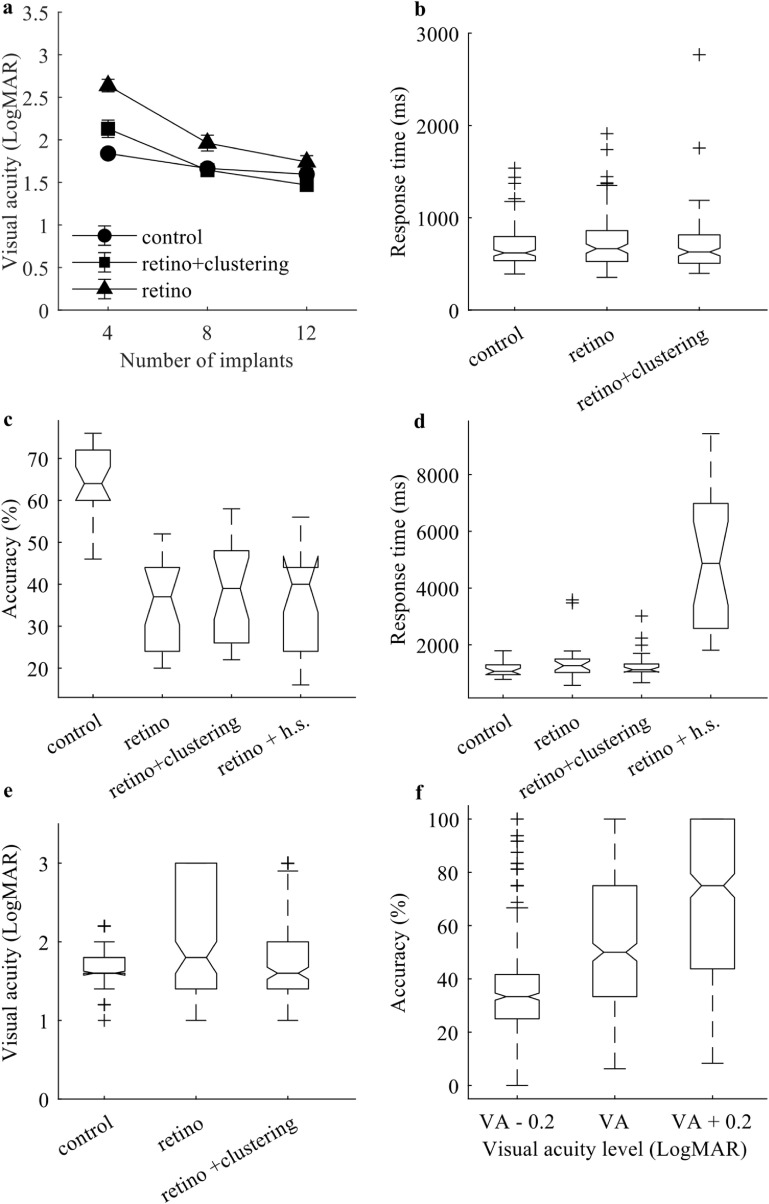
The box plots of different conditions using different three measurements. The first row are results from visual acuity test. The second row are results from object recognition task. For boxplots, the horizontal line in the middle represents the median value. Notches showed the variability of the median between groups. The lower and upper bounds of boxes represent the 25th and 75th percentile, respectively. The whiskers extend towards the maximum and minimum of each group. The ‘+’ signs indicate the number of outliers. (a) and (c) are measured in visual acuity and object recognition accuracy respectively, error bars in (a) represent standard error. Both (b) and (d) are measured in response time. (e) is visual acuity for different presentation methods, an unequal variance was observed. (f) is the result of the validation test. VA represents measured visual acuity.

Because phosphenes are distributed differently between control maps and retinotopic maps, we would expect some differences in visual performances on those maps. This would show whether the utilisation of different maps would affect the measured visual ability. To compare retinotopic maps (*n* = 10 subjects × 5 sessions × 3 implant numbers = 150) with control maps (*n* = 150), we used two-sample *F*-test and found that the variances were not equal between the two groups using both measurements (*p* = 2.33 × 10^−36^ for VA, *p* = 0.0011 for response time), where retinotopic maps had a larger variance than control maps (figures 3(b) and (f)). The linear mixed model showed that both main effects and their interaction are significant (*p* < 0.001). For presentation method, F(2, 432) = 31.431, for number of implants F(2, 432) = 21.43, for interaction of the two predictors, F(4, 432) = 8.42. Conditional pseudo *R* ^2^ = 0.387, marginal pseudo *R* ^2^ = 0.323. The result of *post-hoc* analysis with Bonferroni correction is summarised in table 1. Averaged value and standard error of mean are summarised in figure 3(a). With four and eight implants, control map (presentation method = 0) performed significantly better than retinotopy map (presentation method = 2), but not with 12 implants (*p* < 0.001, *p* = 0.01, *p* = 1.00, respectively). The mean differences also decreased with an increased number of implants (0.80, 0.27, 0.09 LogMAR, respectively). The ANOVA test on the object recognition task showed significant different between groups (F(3, 84) = 40.48, *p* = 2.78 × 10^−6^), the effect size, *η* ^2^ = 0.59, indicating a large effect. *Post-hoc* analysis showed that performance on the control map (64.55% ± 1.73%) is significantly better than the retinotopic map (35.27% ± 2.27%; *p* = 8.07 × 10^−14^; figure 3(c)). Like the VA test, retinotopic maps showed a larger variance, but only significant when measured in response time (*p*= 0.22 for accuracy, *p* = 8.33 × 10^−6^ for response time; figure 3(d)).

**Table 1.** Pairwise comparisons of linear mixed model showed differences between group means for visual acuity test.

Implants number	Presentation method (A)	Presentation method (B)	Mean difference (A − B)	Standard error	df	Sig	95% Confidence interval	
							Upper bound	Lower bound
4	0	1	−.318^a^	.091	432	.002	−.536	−.100
		2	−.798^a^	.091	432	<.001	−1.016	−.580
	1	0	.318^a^	.091	432	.002	.100	.536
		2	−.480^a^	.091	432	<.001	−.698	−.262
	2	0	.798^a^	.091	432	<.001	.580	1.016
		1	.480^a^	.091	432	<.001	.262	.698
8	0	1	.020	.091	432	1.000	−.198	.238
		2	−.270^a^	.091	432	.009	−.488	−.052
	1	0	−.020	.091	432	1.000	−.238	.198
		2	−.290^a^	.091	432	.005	−.508	−.072
	2	0	.270^a^	.091	432	.009	.052	.488
		1	.290^a^	.091	432	.005	.072	.508
12	0	1	.126	.091	432	.498	−.092	.344
		2	−.088	.091	432	1.000	−.306	.130
	1	0	−.126	.091	432	.498	−.344	.092
		2	−.214	.091	432	.057	−.432	.004
	2	0	.088	.091	432	1.000	−.130	.306
		1	.214	.091	432	.057	−.004	.432

For presentation method: control = 0, retinotopy + clustering = 1, retinotopy = 2.

Bonferroni correction was used to adjust *p* value for multiple comparisons.

^a^ The mean difference is significant at the .05 level.

The validation result measured the accuracy around the tested VA (VA + 0.2 LogMAR, VA − 0.2 LogMAR and at VA, as shown in figure 3(f)). The one-way ANOVA test showed that each group was significantly different from the others (*p* = 1.08 × 10^−74^; mean = 0.34 ± 0.19, 0.54 ± 0.27, 0.69 ± 0.28, respectively). This means that accuracy can be used to differentiate visual stimuli at different VA, and it is around the threshold value (0.5) when tested at VA.

### 3.2. Clustering and head scanning improved performance

Because phosphenes cluster into groups on retinotopic maps due to the organisation of visual cortex, we wanted to test if using a pre-processing algorithm or allowing head scanning could improve visual performance. For the VA test, the two-sample *F*-test showed that the clustering algorithm reduced the variance of VA and helped participants achieve more consistent performance (*p* = 0.043; figure 3(e)). For subject response time, the clustering method reduced response time on average (figure 3(b)), and the two-sample *F*-test showed that the variance between the two groups was not statistically significant (*p* = 0.95; figure 3(b)). The pairwise comparisons in table 1 showed that clustering algorithm (presentation method = 1) improved performance on retinotopy maps (presentation method = 2) significantly with 4 and 8 implants, but not with 12 implants. The mean differences also decreased with increasing number of implants (0.48, 0.29, 0.21 LogMAR, respectively). Moreover, clustering algorithm can bring VA to a level equivalent to control map with 8 and 12 implants (*p* = 1.00, *p* = 0.50, respectively), and decrease the mean difference with 4 implants (mean difference = 0.32 LogMAR with clustering, 0.80 LogMAR without clustering). For the object recognition task, although the clustering algorithm (37.82% ± 2.43%) improved performance on retinotopic maps in latter sessions, the improvement is not significant (*p* = 0.85). Since we only test object recognition with 12 implants, this is consistent with the result of VA test, where the benefit of the clustering algorithm decreased as the number of implants increased. Similarly, head scanning (35.91% ± 2.38%) does not improve performance significantly on retinotopic maps on average (*p* = 1.00). It is worth noticing that both the clustering algorithm and head scanning improved performance when evaluated using median value (figure 3(c)), which indicated that the two methods are likely to work on some phosphene maps but not others.

### 3.3. Metrics predict visual performance

We then tested if we could compute metrics that accurately measure the spatial characteristics of phosphene maps, which in turn could be used to predict participants’ visual ability without the need for time-consuming and costly psychophysics testing. We computed three different metrics based on phosphene map properties and visual stimuli properties.

Phosphene ‘map complexity’ was computed using Chen and Sundaram’s method but excluding the trace distance as this is less relevant when presenting visual stimuli on a phosphene map [59]. The complexity consists of three parts, global distance entropy, local angle entropy, and perceptual smoothness. Global distance entropy is computed by the histogram of distance from points to the shape centre. Local angle entropy is computed by the histogram of neighbour points’ angles. The perceptual smoothness of a shape is computed by averaging the smoothness of each point, where the smoothness is determined by the local angle. The smoothness is 0 when the angle is *π* and 1 when the angle is 0. Mathematical details are provided in [59]. This metric measures how a set of points is distributed in a 2D space. For example, a circle would have low complexity, a polygon with noise would have medium complexity and a set of random points would have high complexity. The value was computed for each phosphene map.

‘Mutual information’ was computed between phosphene maps and images of ‘E’, which would have different sizes and orientations. We first padded images to make they have the same size. Then we derived the marginal and joint probability mass function (PMF) of each image based on the histogram of the image. Computation was sped up by excluding extremely small values (less than 10^−12^). Mutual information (*I*) is computed using equation (5), where *P_XY_* is the joint PMF, and *P_X_, P_Y_* is the PMF for each image

\begin{equation*}I = {\text{ }}\mathop {\mathop \sum \nolimits}\limits_{y \in Y} \mathop {\mathop \sum \nolimits}\limits_{x \in X} {P_{XY}}\log \left( {{P_{XY}}/{P_X}{P_Y}} \right).\end{equation*}

We averaged the *I* within each condition. Among all grating acuity test results, only one of them is 2.9 LogMAR, while all the other test results showed a VA greater than 2.9 LogMAR. Hence, grating acuity was excluded from this analysis.

‘Area’ was the area underneath each phosphene map. Since we have phosphenes disperse away from the main clusters, in contrast to the convex hull, we allow the boundary to shrink towards the interior of the hull. We used MATLAB boundary function and shrink factor *s* was set to 1 to get a compact boundary.

**Figure 4.**
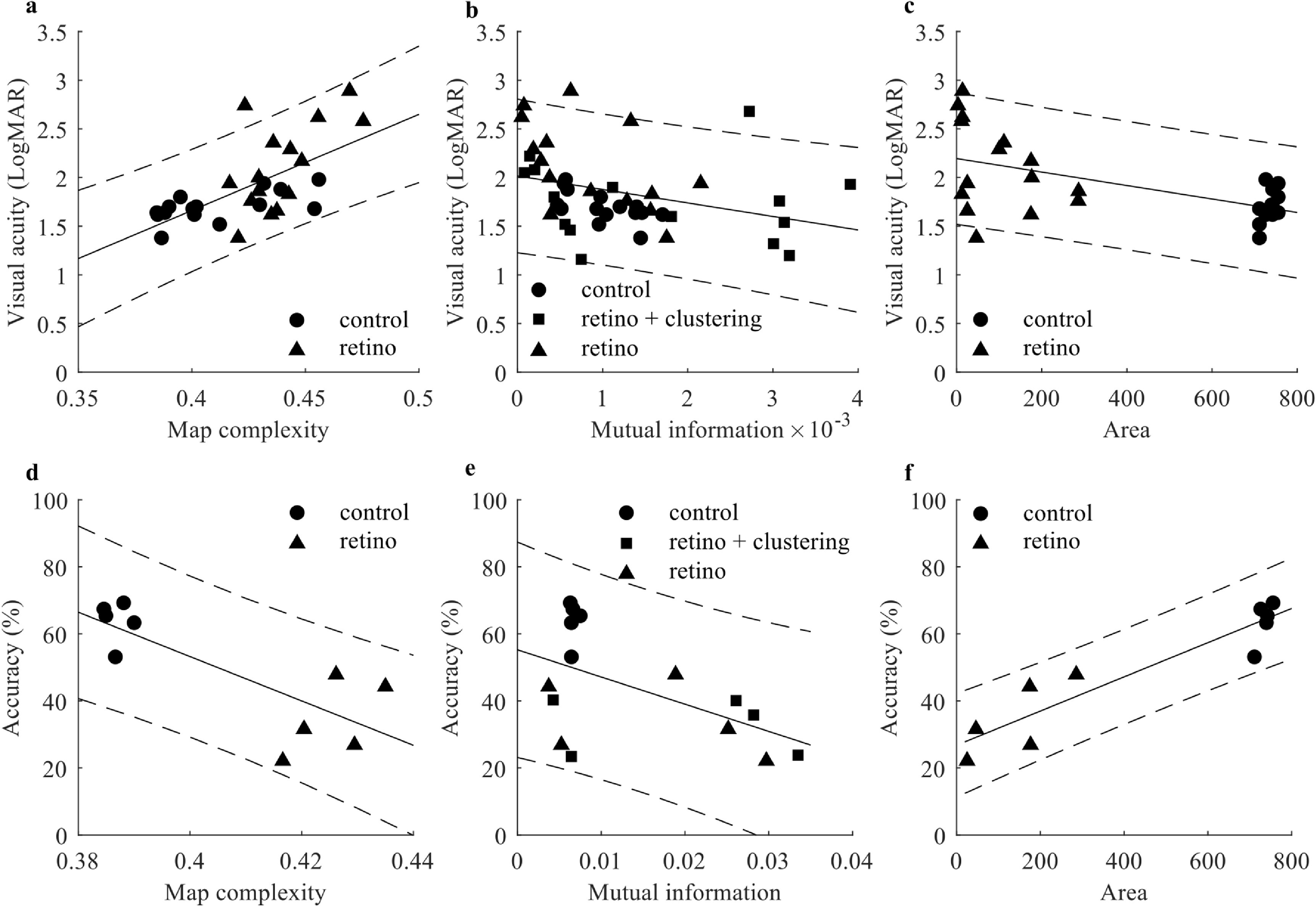
Metrics used to predict participants’ visual performance. First row are results from visual acuity test; second row are results from object recognition task. The solid line is the fit line between each metric and visual acuity. The two dashed lines represent the 95% prediction interval. (a) Map complexity against visual acuity (*R* = 0.65, *p* = 1.07 × 10^−4^, *n* = 30). (b) Mutual information against visual acuity (*R* = −0.33, *p* = 0.026, *n* = 45). (c) Area against visual acuity (*R* = −0.58, *p* = 6.89 × 10^−4^, *n* = 30). (d) Map complexity against accuracy (*R* = −0.79, *p* = 6.5 × 10^−3^, *n* = 10). (e) Mutual information against accuracy (*R* = −0.54, *p* = 0.040, *n* = 15). (f) Area against accuracy (*R* = 0.93, *p* = 8.67 × 10^−5^, *n* = 10).

A linear regression model was computed to fit between each metric and VA for each condition as shown in figure 4. Pearson correlation was calculated to characterise the strength of these relationships. All three metrics, ‘map complexity’, ‘mutual information’ and ‘area’ showed a significant correlation with VA (*p* = 1.07 × 10^−4^, 0.026, 6.89 × 10^−4^, respectively) and accuracy (*p* = 6.5 × 10^−3^, 0.04, 8.67 × 10^−5^, respectively). As shown in figure 4, the ‘map complexity’ is positively correlated with VA in LogMAR (*R* = 0.65) and negatively correlated with accuracy in the object recognition task (*R* = −0.79). This means that a less complicated and more regular phosphene map is more likely to provide better vision. ‘Mutual information’ is negatively correlated to both VA (*R* = −0.33) and accuracy (*R* = −0.54). This indicated that while the VA test benefits from more phosphenes to represent stimuli stroke, while object recognition task needs to present the differences between stimuli, hence high mutual information between stimuli and phosphene map may not be beneficial. ‘Area’ is negatively correlated to VA (*R* = −0.58) and positively to accuracy (*R* = 0.93). This highlights the benefit of having large visual field coverage in prosthetic vision.

To test the size of these correlations and select the best predictor, for each pair of coefficients that is significant we took the absolute value and used Fisher transformation to get *z*-scores. For VA tests, effect size does not differ significantly among all three metrics. However, ‘area’ has significantly larger effect sizes than the others in the object recognition task (*p* = 0.02 when compared with complexity, *p* = 1.32 × 10^−5^ when compared with mutual information). Since the prediction provided ‘mutual information’ is task-specific, we hypothesised that ‘area’ and ‘complexity’ could predict the performance of a map without running psychophysics experiments on participants.

### 3.4. Clustering and head scanning improved performance over multiple sessions

**Figure 5.**
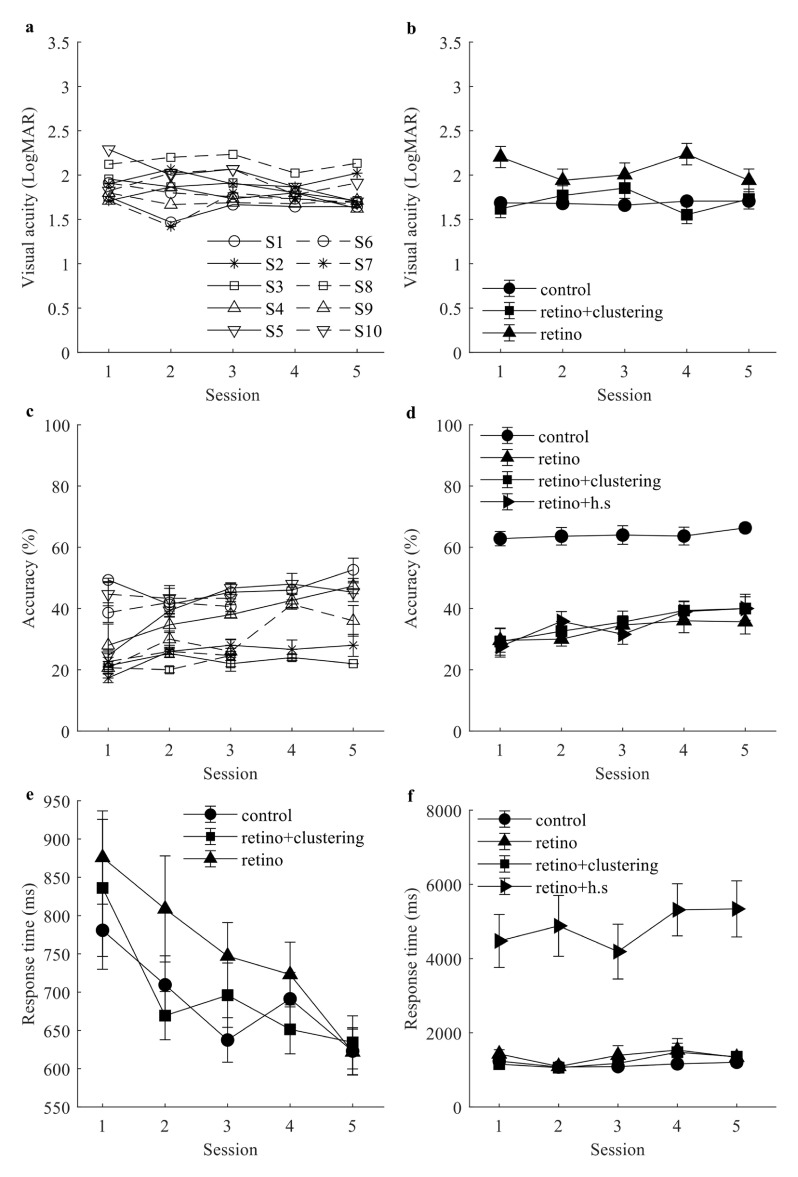
The visual acuity and response time from validation tests across five sessions, error bars are standard errors. (a) and (c) is the learning curve for each subject using visual acuity and object recognition accuracy, each combination of shape and line represents a subject. Error bars are suppressed in panel (a), which make it hard to present mean values. (b) and (d) are learning curves measured for each condition using visual acuity and object recognition accuracy. (e) The learning curves were measured for each condition using response time for visual acuity test. (f) The learning curves were measured for each condition using response time for object recognition task.

Rehabilitation and training are key steps for recipients after being implanted with cortical visual prostheses [60]. Hence, we would like to investigate if participants’ visual ability improved over five sessions. The learning curves observed in VA and response times are shown in figure 5. We averaged across the number of implants and presentation methods to observe the learning curve for each subject (figures 5(a) and (c)). We also averaged across subjects and the number of implants to see if the performance differed among presentation methods through five sessions (figures 5(b) and (d)–(f)).

For all subjects, VA fluctuated between 1.4 LogMAR and 2.3 LogMAR over the five sessions (figure 5(a)). All subjects have an average VA above 1.7 LogMAR for the first session, and seven out of ten subjects’ performances converge between 1.5 and 1.7 LogMAR for the last session. This shows that overall, most subjects’ performance improved over time, but the improvement was not steady across five sessions. This is likely due to the use of retinotopic maps, which are harder to interpret, as shown in figure 5(b). In figure 5(b), the control condition had the most stable performance over the five sessions while using retinotopic maps resulted in worse VA and more unstable performance. The application of the clustering algorithm improved the VA similar to control conditions (as shown in figures 3(e) and (b); table 1), but it still fluctuated over the five sessions. Similar results were observed in the object recognition task. Among six participants who completed five sessions, all of them showed an overall improvement, and three of four participants who completed three sessions showed overall improvement (figure 5(c)). On average, the control condition did not change much across five sessions, while the performance for all three other conditions improved (figure 5(d)). Head scanning improved the most (from 27.6% to 40%), followed by clustering conditions (from 29.2% to 40%), while the retinotopy condition improved the least (29.6%–35.67%).

The response time for different conditions decreased over five sessions and showed a trend of convergence after five sessions in the VA test (figure 5(e)). For the control condition, response time started at 781 ± 51 ms and ended up at 623 ± 31 ms. For the retinotopy conditioning, the response time started at 876 ± 61 ms and ended up at 622 ± 30 ms. When the clustering algorithm is used on retinotopic maps, the response time started at 836 ± 90 ms and ended up at 634 ± 35 ms. We can see that the response time for the control condition seemed to reach the proficient level faster and start to fluctuate after the third session, while response time for the other two conditions almost steadily decreased through five sessions. Response times for object recognition tasks did not vary a lot except for the head scanning condition. Participants spent more time scanning the stimuli in later sessions, which may be one of the reasons that head scanning improved performance the most across five sessions.

## 4. Discussion

### 4.1. Participants perform better on spatially regular maps than retinotopic maps

We found that participants performed better on the control maps than on brain-derived retinotopic maps. In addition, performance on control maps is more consistent (figures 3(e), (c) and 5(b), (d)). With the same number of phosphenes, control maps tend to cover larger visual fields and more evenly than brain-derived maps. This means when we increase the phosphene number, the total visual field covered does not change significantly but the phosphene density increases. On the contrary, with a limited number of phosphenes, retinotopic maps tend to cluster together in a small region. When we increase the number of phosphenes, the coverage of the visual field also tends to increase. In comparison, control maps are more likely to present visual stimuli without cropping, regardless of phosphene number. If we superimpose visual stimuli with phosphene maps, control maps were more likely to cover visual stimuli area than retinotopic maps. More phosphenes on control maps will help present more details but as long as we have enough phosphenes, control maps can capture the overall feature.

As expected, there was significant variability in the structure and retinotopy of the visual cortices examined, which corresponded to variability in implant placement, and ultimately the regions that had phosphene coverage in the generated maps. The previous study has shown that participants’ performance decreased with increasing irregularity of phosphene maps and decreasing coverage ratio [34]. This is consistent with our finding that fewer phosphenes and irregularity of retinotopic maps may lead to poorer performance.

Based on phosphene maps derived from previous stimulation studies in humans and primates [12, 41], our retinotopic maps are closer to those maps than the control maps. Our results suggest that previous simulation studies may overestimate the visual ability that implants can provide for the recipients [18, 35]. This study shows that it is necessary to take into consideration implant placement and the constraints around neurosurgery and device hardware. As it is very difficult to increase electrode and hence phosphene numbers, with a push to already implant as many electrodes as feasible, the next logical optimisation is to improve how we decide on where to implant devices which we will discuss in the next two sections.

### 4.2. Predictability of participant performance from phosphene maps

A quick evaluation of the expected phosphene maps in the clinical setting may greatly improve patient outcomes if we can rule out inappropriate implant placement. Hence, we computed four metrics to test if we can capture the underlying spatial characteristics that are the key to a functional visual experience, to allow evaluation of how ‘good’ a phosphene map is, and what kind of characteristics are needed to make it ‘useable’. All three metrics we computed demonstrate a significant correlation with VA. The ‘area’ appears to have the strongest correlation with VA and object recognition performance, followed by ‘complexity’ and ‘mutual information’. This confirms previous findings where visual field size plays a key role in prosthetic vision [20].

A benefit of ‘complexity’ and ‘area’ is that while ‘mutual information’ is computed based on how we present visual stimuli on phosphene maps, ‘complexity’ and ‘area’ are solely dependent on the phosphene map. This makes it a quick and easy measurement without knowing the visual stimuli. In the actual application of prosthetic vision, the visual stimuli will be more diverse than just letters and simple patterns. Moreover, the number of implants is often not a parameter that can be easily changed and is fixed based on a clinical trial, so the number of phosphenes is usually limited. In this case ‘complexity’ and ‘area’ are more useful metrics likely to capture the difference between maps, which can help us optimise other parameters, such as implant placement. While a dense phosphene map is good for reading, a phosphene map that covers a larger visual angle may be better for navigation tasks [20].

‘Mutual information’ may be a better way if we have both visual stimuli and a phosphene map at hand. This also explained the fact that the relationship between ‘mutual information’ and performance is task-specific (figures 5(b) and (e)). On the one hand, for tasks that required detailed information, we may want more mutual information between the phosphene map and visual stimuli, while for tasks that required recognising overall features, we want less mutual information so participants can discriminate the differences. To do this we can set a threshold for this value and predict if visual stimuli can be represented on a given phosphene map reasonably well. Hence, computing mutual information may inform recipients what kind of tasks they are most likely to benefit from.

In clinical settings, these metrics might be a quick pre-surgery process to help evaluate how useable a derived phosphene map would be. The measurement of a phosphene map’s inherent characteristics might be a more efficient way to evaluate a phosphene map. This can help us narrow down options for implant placement before hiring sighted participants to perform psychophysics experiments. If the resulting map has a limited number of phosphenes or is overly complex, we may need to be cautious about proceeding with surgical implantation.

### 4.3. Clinical applications of the current simulation paradigm

**Figure 6.**
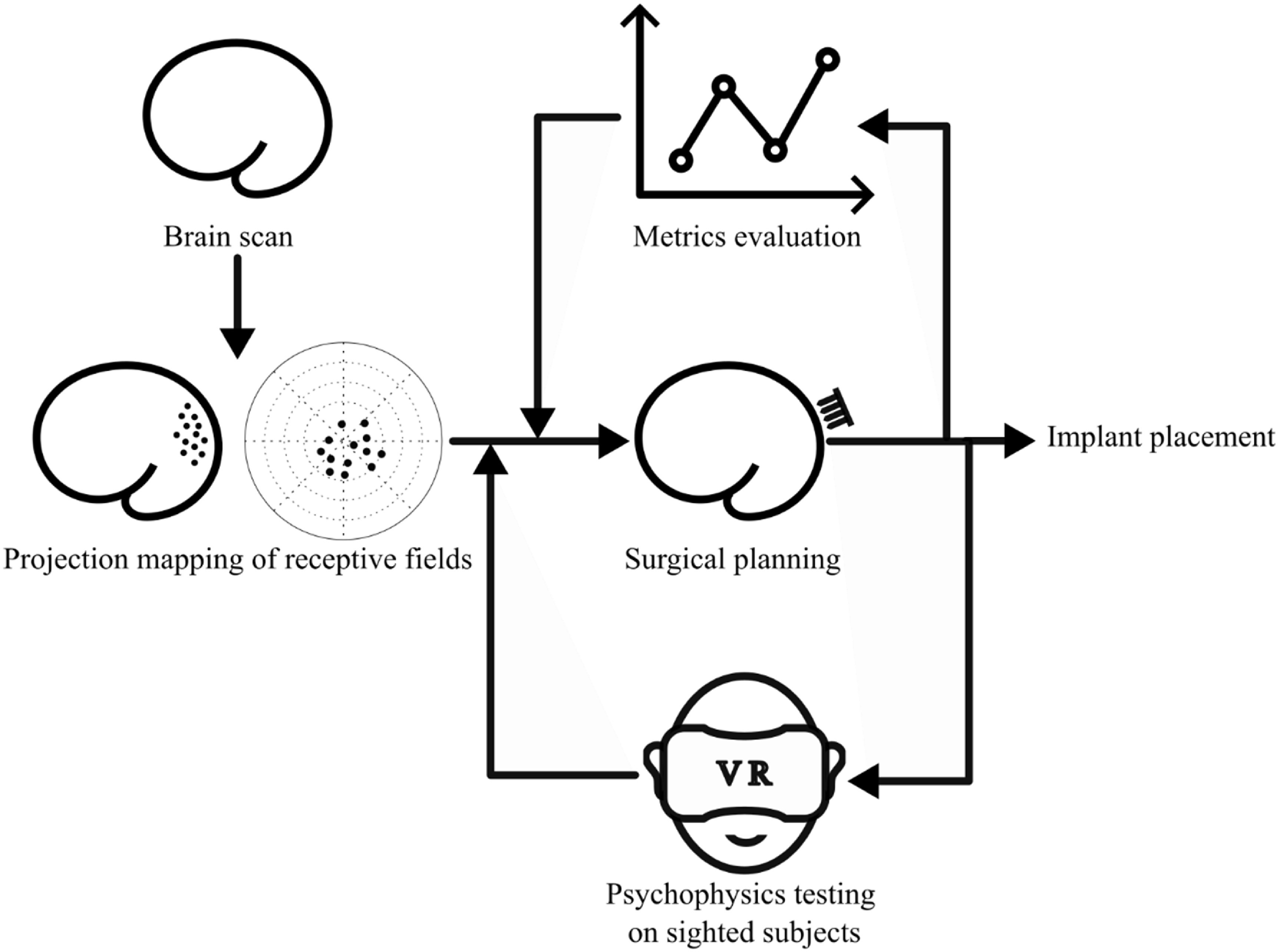
The planning process for a cortical visual prosthesis. When a potential recipient comes in, we first get their cortex surface topology via a structural MRI. A receptive field map of visual cortex can be obtained in two ways, (1) for acquired blind subjects, we could predict the receptive field based on cortex surface topology, using the retinotopy dataset as the sample space; and (2) for congenitally blind subjects, we could get the retinotopic map using DTI seeded from LGN, which will predict the map via optic tracts [61]. Once we derived retinotopic maps, we could use the simulation paradigm proposed in this study to predict the phosphene map. Phosphene maps can be first evaluated using metrics we proposed to select candidate options for practical implant placement. We can then test those options by running psychophysics experiments on sighted participants using a virtual reality headset. Once the performance reaches a promising level, we can proceed to implant placement.

Currently, we can either use an MRI scan to acquire cortical surface geometry before surgery or use transcranial magnetic stimulation to decide if certain regions can elicit phosphenes [12, 52]. However, there is no current method to evaluate a subject’s visual ability with a cortical visual prosthesis before implantation or with different implant locations. Hence, we propose a new process that incorporates our simulation paradigm into the surgical planning of cortical visual prostheses, as shown in figure 6. Before a subject is implanted with a cortical visual prosthesis, a structural MRI scan can be conducted to acquire the shape of their cortical surface. Putative receptive fields in the visual cortex can then be estimated using diffusion tensor imaging (DTI) seeded from the lateral geniculate nucleus [61]. Finally, the surgery and implant locations can be planned, and the simulation paradigm described in this paper can be used to derive phosphene maps. Then we can use those metrics we computed to find candidate implant locations that are more likely to give us a usable phosphene map. After that, we can hire sighted participants to verify the correct choice of maps and implant locations through a series of psychophysics experiments. These could include reading, object recognition, object manipulation and navigation.

### 4.4. Attempts to improve visual performance on retinotopic maps

Given the poor performance on retinotopic maps, we attempted to improve performance by introducing clustering and head scanning into our simulation paradigm. We first tried to shift the stimuli centre to the phosphene map centroid, allowing us to present visual stimuli in phosphene-dense regions. We found that using a clustering algorithm improved the average performance and consistency in VA tests significantly with four and eight implants (figures 3(a) and (b)). Even though this shifts the visual stimuli away to a more peripheral region, this increases the number of neighbouring phosphenes that can be used to present visual stimuli.

Despite the benefit of clustering, the overall VA was still low, and the improvement is not significant for the object recognition task. In our experiment design, two parameters of the algorithm were kept constant to reduce unnecessary variability between conditions. If we aimed for an optimised output, we could tune these parameters based on the phosphene map and the size of the visual stimulus we want to present. The current algorithm only separates the majority of phosphenes from outliers, which is sufficient for a VA test, in which we only showed single letters or simple patterns on phosphene maps. In a more complex task such as object recognition, the benefit was less obvious and took more sessions to appear (figures 3(c) and 5(d)).

This highlights the need of extracting spatial characteristics that are unique for each phosphene map and optimising the rendering output. A lot of effort has been made to extract features from the environment [28, 32, 33], but not many studies have tried to address the variability of phosphene maps, and how we can present visual stimuli adaptively. Our results indicate that we might need to shift our focus from feature extraction to rendering. There are a lot of existing tools from computer vision for feature extraction, whereas there are limited techniques to render those features on a phosphene map.

Head scanning in object recognition tasks provided progressive advantages as we ran more sessions (figure 5(d)). However, on average this improvement was not significant (figure 3(c)). A previous study quantitatively showed that head scanning could improve performance in VA tests [57], but we could not generalise this finding in object recognition tasks. A key difference between our experiment and the previous study is the phosphene map we used. It would be harder for irregular phosphene maps to provide continuous perception in head scanning. Since retinotopic phosphene maps have their unique boundary, this may interfere with the characteristics of given stimuli and makes it harder to discriminate among them. Another major improvement compared to the previous study is including eye tracking in the simulation paradigm. Although previous studies encourage participants to fixate at the centre there is no guarantee that eye movement was eliminated. The disassociation between head and eye position may make it harder to implement head scanning during the tasks.

When applying these methods to an actual implant recipient, a potential problem is that visual stimuli may not be present at the centre of the visual field or align with the physical location. This is less of a problem in reading tasks, where recipients may sit still, but is a challenge if a recipient needs to interact with the environment, such as walking around and manipulating objects. In these cases, the misalignment between spatial locations and perceived visual field locations may cause confusion, however there is evidence that with training, participants are able to negate this effect [18].

Apart from training recipients, the rendering method can also be task oriented [25]. For some tasks, such as reading, we need enough phosphenes to present each letter [54]. While for other tasks, such as object recognition or navigation, we want to maximise the visual field that the phosphenes cover so recipients can quickly scan their environment [20]. Alternatively, head scanning could be used to compensate for eye movement. However, participants would need to wear a headset with eye-tracking and head-tracking [36, 62].

### 4.5. Different experiments demonstrated different learning curves

Several studies have shown that participants’ visual ability can improve with appropriate training after being implanted with a visual prosthesis [12, 63, 64]. VA showed a slight improvement when the last session was compared against the first session (figure 5(a)). Participants also responded faster, as response time also showed a decreased trend based on our result (figure 5(e)). However, participants’ performance fluctuated a lot throughout the five sessions.

For the VA test, most of the fluctuation comes from the retinotopy groups (figure 5(b)) while participants’ performance was less consistent on retinotopic maps (figures 3(b) and (e)). The inconsistency may result in fluctuation and hence makes the learning effect less obvious (figures 5(a) and (b)). Two reasons may result in an unobvious learning effect. First, we did not include feedback in our experiment as we did not want participants to learn from spurious cues. Rather than recognising the actual orientation, participants may respond correctly just by memorising the differences between visual stimuli. Hence instead of measuring VA, we would be measuring participants’ ability to memorise and discriminate between different images, which would exaggerate measured visual ability.

Second, because of the monitor size and resolution, the range of discrete measurement is limited between 0.6 and 2.9 LogMAR, with a step of about 0.2 LogMAR. We observed that few valid test results were obtained by grating acuity. In the BRVT paradigm, the grating acuity test was used to test those maps that failed the single tumbling E test. Most of the maps that required a grating acuity test are retinotopic maps with low phosphene numbers. Due to limited visual field coverage of retinotopic maps, and the clustered distribution of phosphenes, many grating stimuli could not be fully presented. This may cause some changes in VA that cannot be captured finely, which also results in large fluctuation in the retinotopy group. This result highlights the importance of capturing realistic phosphene distribution and may change the way we test prosthetic vision. Since the phosphenes were not distributed evenly in the visual field, some conventional VA tests, such as the grating acuity test, may not be suitable for prosthetic vision.

For object recognition tasks, nine out of ten participants showed improvement when comparing their accuracy of the last session to the first session (figure 5(b)). While the control condition stayed at the same level across five sessions, all three other conditions showed noticeable improvement. Among the three retinotopy conditions, the retinotopy group was saturated after the third session. The head scanning demonstrated the most improvement along with an increase in response time (figure 5(f)). A similar pattern of accuracy and response time showed the potential benefit of head scanning may be further extended if participants could spend more time with the given object. Our result suggests that both clustering and head scanning might require longer training periods to reach a saturated performance level that will show a significant improvement on retinotopic maps. In the eventual visual prosthesis, a gaze compensation may accelerate this process and provide a more intuitive visual experience [36, 62]. The differences between VA and object recognition task highlight the need of applying more complex tasks to evaluate prosthetic vision rather than relying on VA tests alone.

### 4.6. Future applications of brain-derived phosphene simulation paradigms

The proposed simulation paradigm can be easily adapted to various hardware configurations and investigate different aspects of cortical prosthetic vision. Another feature we can investigate using the current simulation paradigm is the temporal feature of phosphenes. In our study, we limited the exposure time to visual stimuli and restricted participants’ head and eye movement to minimise the temporal effect of phosphenes. However, if we run more complex psychophysics tasks, we need to consider how phosphenes can change over time in the simulation paradigm. Although phosphenes generally appear and disappear in time with stimulation [3, 5, 8, 11, 12], other spatiotemporal features of phosphenes appeared to be complicated in cortical visual prostheses. For example, consecutive stimulation of the same site might decrease phosphene brightness [5, 8, 65], but this is not replicated in a more recent human trial [12]. Moreover, when stimulating multiple electrodes simultaneously, phosphenes would become brighter and clearer [12]. Phosphenes could also drift between different stimulation sessions [12]. We tried to minimise the effect of temporal instability by limiting the visual stimuli to 50 ms, but if we want to test more complex tasks, such as navigation, and investigate the potential benefit of head scanning, these temporal features should be considered.

After the implantation of a cortical prosthesis, a phosphene mapping procedure is usually conducted to determine phosphene locations [12, 66, 67]. However, there could be some errors or mismatches between indicated and actual phosphene locations. Previous studies have shown that loss of retinotopic information can result in significantly poor performance in a VA test [68]. In cortical visual prostheses, these errors usually vary between individuals as well as stimulation parameters [41, 66]. Current simulation paradigm can be used to explore how the number of mismatched phosphenes, or error level would affect participants’ performance in cortical visual prostheses. This can also be used to test how robust a given image processing algorithm is in the presence of phosphene mapping error. Moreover, a longer study where head scanning is allowed can investigate if participants can adapt to these errors and improve their visual ability over time.

## 5. Conclusion

We used an existing retinotopy dataset to generate a more realistic phosphene map for cortical prosthetic vision. We found that visual performance is worse in brain-derived retinotopic maps when compared with control maps. This may indicate that visual ability with implants has been overestimated in previous simulation studies. This is the first paradigm that can realistically simulate the visual experience of cortical prosthetic vision. Further, enabling head scanning and a simple algorithm looking at the spatial characteristics phosphene map showed the potentiality of improving visual ability. In future clinical practice, this simulation paradigm may facilitate the decision-making process of planning implant locations before surgery, which will help to optimise the outcome of cortical visual prostheses.

## Data Availability

The data cannot be made publicly available upon publication because they are owned by a third party and the terms of use prevent public distribution. The data that support the findings of this study are available upon reasonable request from the authors.

## References

[1] Luo Y H L, da Cruz L (2016). The Argus^®^ II retinal prosthesis system. Prog. Retin. Eye Res..

[2] Pezaris J S, Reid R C (2007). Demonstration of artificial visual percepts generated through thalamic microstimulation. Proc. Natl Acad. Sci..

[3] Brindley G S, Lewin W S (1968). The sensations produced by electrical stimulation of the visual cortex. J. Physiol..

[4] Dobelle W H, Mladejovsky M G, Girvin J P (1974). Artificial vision for the blind: electrical stimulation of visual cortex offers hope for a functional prosthesis. Science.

[5] Schmidt E M, Bak M J, Hambrecht F T, Kufta C V, O’Rourke D K, Vallabhanath P (1996). Feasibility of a visual prosthesis for the blind based on intracortical microstimulation of the visual cortex. Brain.

[6] Italiano M L, Guo T, Lovell N H, Tsai D (2022). Improving the spatial resolution of artificial vision using midget retinal ganglion cell populations modeled at the human fovea. J. Neural Eng..

[7] Hallum L E, Dakin S C (2021). Retinal implantation of electronic vision prostheses to treat retinitis pigmentosa: a systematic review. Transl. Vis. Sci. Technol..

[8] Dobelle W H, Mladejovsky M G (1974). Phosphenes produced by electrical stimulation of human occipital cortex, and their application to the development of a prosthesis for the blind. J. Physiol..

[9] Meikle S J, Hagan M A, Price N S C, Wong Y T (2022). Intracortical current steering shifts the location of evoked neural activity. J. Neural Eng..

[10] Allison-Walker T, Hagan M A, Price N S C, Wong Y T (2021). Microstimulation-evoked neural responses in visual cortex are depth dependent. Brain Stimul..

[11] Beauchamp M S, Oswalt D, Sun P, Foster B L, Magnotti J F, Niketeghad S, Pouratian N, Bosking W H, Yoshor D (2020). Dynamic stimulation of visual cortex produces form vision in sighted and blind humans. Cell.

[12] Fernández E (2021). Visual percepts evoked with an intracortical 96-channel microelectrode array inserted in human occipital cortex. J. Clin. Invest..

[13] Bourkiza B, Vurro M, Jeffries A, Pezaris J S (2013). Visual acuity of simulated thalamic visual prostheses in normally sighted humans. PLoS One.

[14] Cha K, Horch K W, Normann R A, Boman D K (1992). Reading speed with a pixelized vision system. J. Opt. Soc. Am. A.

[15] Cha K, Horch K W, Normann R A (1992). Mobility performance with a pixelized vision system. Vis. Res..

[16] Endo T, Hozumi K, Hirota M, Kanda H, Morimoto T, Nishida K, Fujikado T (2019). The influence of visual field position induced by a retinal prosthesis simulator on mobility. Graefe’s Arch. Clin. Exp. Ophthalmol..

[17] Ho E, Boffa J, Palanker D (2019). Performance of complex visual tasks using simulated prosthetic vision via augmented-reality glasses. J. Vis..

[18] Srivastava N R, Troyk P R, Dagnelie G (2009). Detection, eye-hand coordination and virtual mobility performance in simulated vision for a cortical visual prosthesis device. J. Neural Eng..

[19] Sanchez-Garcia M, Martinez-Cantin R, Bermudez-Cameo J, Guerrero J J (2020). Influence of field of view in visual prostheses design: analysis with a VR system. J. Neural Eng..

[20] Thorn J T, Migliorini E, Ghezzi D (2020). Virtual reality simulation of epiretinal stimulation highlights the relevance of the visual angle in prosthetic vision. J. Neural Eng..

[21] Van Rheede J J, Kennard C, Hicks S L (2010). Simulating prosthetic vision: optimizing the information content of a limited visual display. J. Vis..

[22] De Ruyter Van Steveninck J, Güçlü U, Van Wezel R, Van Gerven M (2022). End-to-end optimization of prosthetic vision. J. Vis..

[23] Irons J L, Gradden T, Zhang A, He X, Barnes N, Scott A F, McKone E (2017). Face identity recognition in simulated prosthetic vision is poorer than previously reported and can be improved by caricaturing. Vis. Res..

[24] Li H, Su X, Wang J, Kan H, Han T, Zeng Y, Chai X (2018). Image processing strategies based on saliency segmentation for object recognition under simulated prosthetic vision. Artif. Intell. Med..

[25] Lozano A, Suarez J S, Soto-Sanchez C, Garrigos J, Martinez-Alvarez J J, Ferrandez J M, Fernández E (2020). Neurolight: a deep learning neural interface for cortical visual prostheses. Int. J. Neural Syst..

[26] Sanchez-Garcia M, Martinez-Cantin R, Guerrero J J, Wang Y (2020). Semantic and structural image segmentation for prosthetic vision. PLoS One.

[27] White J, Kameneva T, McCarthy C (2022). Vision processing for assistive vision: a deep reinforcement learning approach. IEEE Trans. Hum. Mach. Syst..

[28] Zhao Y, Lu Y, Tian Y, Li L, Ren Q, Chai X (2010). Image processing based recognition of images with a limited number of pixels using simulated prosthetic vision. Inf. Sci..

[29] Vurro M, Crowell A M, Pezaris J S (2014). Simulation of thalamic prosthetic vision: reading accuracy, speed, and acuity in sighted humans. Front. Hum. Neurosci..

[30] Dagnelie G, Keane P, Narla V, Yang L, Weiland J, Humayun M (2007). Real and virtual mobility performance in simulated prosthetic vision. J. Neural Eng..

[31] Chen S C, Hallum L E, Lovell N H, Suaning G J (2005). Visual acuity measurement of prosthetic vision: a virtual-reality simulation study. J. Neural Eng..

[32] Parikh N, Itti L, Humayun M, Weiland J (2013). Performance of visually guided tasks using simulated prosthetic vision and saliency-based cues. J. Neural Eng..

[33] Van Steveninck J D R, Van Gestel T, Koenders P, Van Der Ham G, Vereecken F, Güçlü U, van Gerven M, Güçlütürk Y, van Wezel R (2022). Real-world indoor mobility with simulated prosthetic vision: the benefits and feasibility of contour-based scene simplification at different phosphene resolutions. J. Vis..

[34] Lu Y, Kan H, Liu J, Wang J, Tao C, Chen Y, Ren Q, Hu J, Chai X (2013). Optimizing Chinese character displays improves recognition and reading performance of simulated irregular phosphene maps. Investigative Ophthalmol. Vis. Sci..

[35] Fehervari T, Matsuoka M, Okuno H, Yagi T (2010).

[36] Paraskevoudi N, Pezaris J S (2021). Full gaze contingency provides better reading performance than head steering alone in a simulation of prosthetic vision. Sci. Rep..

[37] Lowery A J (2017). Monash Vision Group’s Gennaris cortical implant for vision restoration. Artificial Vision.

[38] Niketeghad S, Muralidharan A, Patel U, Dorn J D, Bonelli L, Greenberg R J, Pouratian N (2020). Phosphene perceptions and safety of chronic visual cortex stimulation in a blind subject. J. Neurosurg..

[39] Troyk P R (2017). The intracortical visual prosthesis project. Artificial Vision.

[40] Dougherty R F, Koch V M, Brewer A A, Fischer B, Modersitzki J, Wandell B A (2003). Visual field representations and locations of visual areas V1/2/3 in human visual cortex. J. Vis..

[41] Chen X, Wang F, Fernandez E, Roelfsema P R (2020). Shape perception via a high-channel-count neuroprosthesis in monkey visual cortex. Science.

[42] Bradley D C (2005). Visuotopic mapping through a multichannel stimulating implant in primate V1. J. Neurophysiol..

[43] Caspi A, Barry M P, Patel U K, Salas M A, Dorn J D, Roy A, Niketeghad S, Greenberg R J, Pouratian N (2021). Eye movements and the perceived location of phosphenes generated by intracranial primary visual cortex stimulation in the blind. Brain Stimul..

[44] Benson N C (2018). The Human Connectome Project 7 Tesla retinotopy dataset: description and population receptive field analysis. J. Vis..

[45] Van Essen D C, Smith S M, Barch D M, Behrens T E J, Yacoub E, Ugurbil K (2013). The WU-Minn Human Connectome Project: an overview. NeuroImage.

[46] Wong Y T, Feleppa T, Mohan A, Browne D, Szlawski J, Rosenfeld J V, Lowery A (2019). CMOS stimulating chips capable of wirelessly driving 473 electrodes for a cortical vision prosthesis. J. Neural Eng..

[47] Cha K, Horch K, Normann R A (1992). Simulation of a phosphene-based visual field: visual acuity in a pixelized vision system. Ann. Biomed. Eng..

[48] Murphey D K, Maunsell J H R, Beauchamp M S, Yoshor D (2009). Perceiving electrical stimulation of identified human visual areas. Proc. Natl Acad. Sci..

[49] Wang L, Mruczek R E B, Arcaro M J, Kastner S (2015). Probabilistic maps of visual topography in human cortex. Cereb. Cortex.

[50] Szlawski J, Feleppa T, Mohan A, Wong Y T, Lowery A J (2022). A model for assessing the electromagnetic safety of an inductively coupled, modular brain-machine interface. IEEE Trans. Neural Syst. Rehabil. Eng..

[51] Harvey B M, Dumoulin S O (2011). The relationship between cortical magnification factor and population receptive field size in human visual cortex: constancies in cortical architecture. J. Neurosci..

[52] Bosking W H, Sun P, Ozker M, Pei X, Foster B L, Beauchamp M S, Yoshor D (2017). Saturation in phosphene size with increasing current levels delivered to human visual cortex. J. Neurosci..

[53] Chen S C, Suaning G J, Morley J W, Lovell N H (2009). Simulating prosthetic vision: I. Visual models of phosphenes. Vis. Res..

[54] Chen S C, Suaning G J, Morley J W, Lovell N H (2009). Simulating prosthetic vision: II. Measuring functional capacity. Vis. Res..

[55] Bailey I L, Jackson A J, Minto H, Greer R B, Chu M A (2012). The Berkeley rudimentary vision test. Optom. Vis. Sci..

[56] Ayton L N (2020). Harmonization of outcomes and vision endpoints in vision restoration trials: recommendations from the international HOVER taskforce. Transl. Vis. Sci. Technol..

[57] Chen S, Hallum L, Suaning G, Lovell N (2007). A quantitative analysis of head movement behaviour during visual acuity assessment under prosthetic vision simulation. J. Neural Eng..

[58] Kanagala H K, Krishnaiah V J R (2016). A comparative study of K-means, DBSCAN and OPTICS.

[59] Chen Y, Sundaram H (2005). Estimating complexity of 2D shapes.

[60] Lewis P M, Ackland H M, Lowery A J, Rosenfeld J V (2015). Restoration of vision in blind individuals using bionic devices: a review with a focus on cortical visual prostheses. Brain Res..

[61] Wu W, Rigolo L, O’Donnell L J, Norton I, Shriver S, Golby A J (2012). Visual pathway study using in vivo diffusion tensor imaging tractography to complement classic anatomy. Oper. Neurosurg..

[62] Titchener S A, Shivdasani M N, Fallon J B, Petoe M A (2018). Gaze compensation as a technique for improving hand–eye coordination in prosthetic vision. Transl. Vis. Sci. Technol..

[63] Yue L, Weiland J D, Roska B, Humayun M S (2016). Retinal stimulation strategies to restore vision: fundamentals and systems. Prog. Retin. Eye Res..

[64] Brelén M E, Duret F, Gérard B, Delbeke J, Veraart C (2005). Creating a meaningful visual perception in blind volunteers by optic nerve stimulation. J. Neural Eng..

[65] Rushton D N, Brindley G S (1978). Properties of cortical electrical phosphenes.

[66] Oswalt D (2021). Multi-electrode stimulation evokes consistent spatial patterns of phosphenes and improves phosphene mapping in blind subjects. Brain Stimul..

[67] Stronks H C, Dagnelie G (2011). Phosphene mapping techniques for visual prostheses. Visual Prosthetics.

[68] Petoe M A, McCarthy C D, Shivdasani M N, Sinclair N C, Scott A F, Ayton L N, Barnes N M, Guymer R H, Allen P J, Blamey P J (2017). Determining the contribution of retinotopic discrimination to localization performance with a suprachoroidal retinal prosthesis. Investigative Ophthalmol. Vis. Sci..

